# Retraction: “Diagnostic Classification and Prognostic Prediction Using Common Genetic Variants in Autism Spectrum Disorder: Genotype-Based Deep Learning”

**DOI:** 10.2196/76833

**Published:** 2025-05-06

**Authors:** JMIR Editorial Office

**Affiliations:** 1 JMIR Publications, 130 Queens Quay East, Suite 1100-1102, Toronto, ON, M5A 0P6, Canada, 1 4165832040

The article “Diagnostic Classification and Prognostic Prediction Using Common Genetic Variants in Autism Spectrum Disorder: Genotype-Based Deep Learning” [1] is being retracted due to analytical errors and irreproducibility of the results, which led to the overestimation of predictive value. The issues are detailed by the authors of a letter to the editor [2] and were confirmed during investigation by JMIR Publications.

Authors Wang and Avillach responded and expressed neither agreement nor disagreement with the decision.

## References

[1] Wang H, Avillach P (2021). Diagnostic classification and prognostic prediction using common genetic variants in autism spectrum disorder: genotype-based deep learning. JMIR Med Inform.

[2] Miller C, Portlock T, Nyaga DM, Gamble GD, O’Sullivan JM (2025). Code error in “Diagnostic Classification and Prognostic Prediction Using Common Genetic Variants in Autism Spectrum Disorder: Genotype-Based Deep Learning”. JMIR Med Inform.

